# Interferon-gamma 1b-induced gene expression alters neutrophil function in patients with chronic granulomatous disease

**DOI:** 10.1371/journal.pone.0331657

**Published:** 2025-09-08

**Authors:** Daniel R. Ambruso, Natalie J. Briones, Alexander D. Tran, Bridget Sanford, Christine Childs, Ben Z. Katz, Michael Ellison, Richard B. Johnston, Kenneth L. Jones

**Affiliations:** 1 Department of Pediatrics University of Colorado Denver, Anschutz Medical Campus, Aurora, Colorado, United States of America; 2 Center for Cancer and Blood Disorders, Children’s Hospital Colorado, Aurora, Colorado, United States of America; 3 University of Colorado Cancer Center Flow Cytometry Shared Resource, Anschutz Medical Campus, Aurora, Colorado, United States of America; 4 Northwestern University Feinberg School of Medicine, Department of Pediatrics and Division of Infectious Diseases, Anna and Robert H. Lurie Children’s Hospital of Chicago, Chicago, Illinois, United States of America; 5 National Jewish Health, Denver, Colorado, United States of America; National Institutes of Health, UNITED STATES OF AMERICA

## Abstract

Interferon-gamma (IFN-γ) decreases infections in chronic granulomatous disease (CGD) with variably incomplete restoration of the fundamental CGD defect, converting oxygen to microbicidal oxidants during phagocytosis. We sought to understand other IFN-γ effects that could contribute to protection of CGD patients. We measured neutrophil function, gene expression, and biochemical parameters in nine CGD patients off IFN-γ and 10–12 hours after the first (1st) and fourth (4th) IFN-γ injection. Non-directed motility and bactericidal activity increased after IFN-γ administration; ingestion remained unchanged. Stimulated release of superoxide anion (O_2_^-^) was minimally changed. Treatment decreased expression of 483 genes and increased 386. Expression of eleven genes associated with neutrophil activity was upregulated. Genes not routinely associated with neutrophil function also demonstrated increased expression, including MHCI & II proteins, guanylate-binding proteins, and an enzyme synthesizing a nitric-oxide (NO) synthetase cofactor. CD11b expression, F-actin assembly, and CD 274 antigen were increased after treatment as was NO in neutrophil lysates. Formation of neutrophil extracellular traps after ingestion of staphylococci was increased off IFN-γ compared with controls but moved toward normal after IFN-γ administration. IFN-γ protects against infection in CGD by several mechanisms that could potentially support others with compromised host defense.

**Trial registration:**

ClinicalTrials.gov NCT03548818

## Introduction

Named for their potent ability to interfere with viral infections, interferons (IFNs) are powerful regulators of the immune system [1,2]. Interferon-gamma 1-b (IFN-γ) has the strongest and most diverse effects of these cytokines. Predominantly secreted by CD8 + T cells, CD4 + T helper-1 cells, and natural killer cells, IFN-γ exerts its effects through interaction with the interferon-gamma receptor (IFNGR) and through the canonical JAK-STAT pathway [3]. It modulates expression of multiple genes and produces a variety of neutrophil responses after *in vivo* administration or *in vitro* exposure [3–12].

As neutrophils and macrophages ingest microbes, plasma membrane components of the complex enzyme nicotinamide adenine dinucleotide phosphate (NADPH) oxidase are activated in a “respiratory burst” that converts oxygen to superoxide anion (O_2_^-^) and its microbicidal reactive-oxygen metabolites [13–15]. Chronic granulomatous disease (CGD) is a genetic disorder of the NADPH oxidase system in phagocytic cells [15] that results in a lifetime of life-threatening infections and, in some patients, inflammatory diseases due at least in part to impaired removal of apoptotic neutrophils by macrophages [16].

Early studies suggested that administration of IFN-γ reduced the rate of infection in patients with CGD without an apparent change in respiratory burst activity in neutrophils or monocytes. A randomized controlled trial (RCT) with 128 CGD patients showed a conclusive reduction in serious infections with IFN-γ injection compared with placebo “without significant changes” in production of O_2_^-^ [17]. However, several studies conducted during the timeframe of the RCT described “variant” forms of CGD with decreased but detectable stimulated neutrophil O_2_^-^ production [9–11].

Using an improved assay and a longer assay time, Kuhns et al. detected at least very small amounts of phorbol myristate acetate (PMA)-stimulated respiratory burst activity in all 227 patients examined [12]. Patient survival was strongly associated with the level of residual O_2_^-^ production grouped into quartiles. Our patients exhibited small if any increase in stimulated O_2_^-^ release after IFN-γ that would presumably place them in the lowest quartile of the analysis of Kuhns et al. [12].

While the stimulatory effect of IFN-γ on the respiratory burst in at least most CGD patients variably improves their survival [12], this cytokine effects a variety of genes producing multiple responses that may also promote protection against infection [5]. Prophylactic administration of IFN-γ along with antimicrobial agents remains a mainstay of treatment for the disorder [15,17–21], yet the mechanism(s) by which IFN-γ achieves its clinical effects in CGD are not completely understood.

We have used CGD as an experiment of nature to obtain greater understanding of this centrally important cytokine. We found that therapeutic administration of IFN-γ altered CGD neutrophil gene and protein expression and biochemical and functional responses that could provide non-oxygen-dependent resistance against infection.

## Materials and methods

### Study design

This study was developed to evaluate the *in vivo* effects of IFN-γ on neutrophils from patients with CGD. The protocol was approved by the Scientific Advisory and Review Committee for scientific review at the Colorado Clinical Translational Sciences Institute and then by the University of Colorado COMIRB for human subjects’ review before initiation of research activities. This research was conducted under ethical standards in accordance with the Declaration of Helsinki. We hypothesized that neutrophils developed under the influence of Interferon- gamma-1b *in vivo* would display enhanced functional and biochemical activities related in large part to the transcriptional activation effects of this cytokine even though the basic genetic defect was not significantly altered. The primary objective of this study was to investigate the relationship between the functional effects of IFN-γ and the transcriptional activity of this cytokine in patients with CGD. The secondary objectives were to characterize how the biochemical pathways and IFN-γ induced changes produced by gene expression activity enhanced the function of neutrophils and provided for the clinical benefits observed for the patients.

### Study approval

Patients with a molecular or genetically defined variant of CGD without infection, inflammation, or other medical complication in the previous month were eligible for enrollment into the study. Participants were recruited locally through patient rosters in the Immunohematology Clinic at Children’s Hospital Colorado and a second out-of-state site through COMIRB-approved flyers distributed at the site. The out-of-state IRB ceded responsibility for the protocol to the COMIRB. Each subject or their parent gave written informed consent during a face-to-face meeting with the principal investigator in a confidential examining room prior to inclusion in the study. The study protocol (COMIRB Protocol #17–1676), initially registered on (01/03/2018) was approved by the COMIRB at the University of Colorado, Anschutz Medical Campus. Our laboratory is certified by the Institutional Biosafety Committee (Protocol #1189). The protocol was also registered on clinicaltrials.gov as NCT03548818 (first posted 07/06/2018), Role of Interferon-gamma 1-b (IFN-γ) on Cells of the Innate Immune System in Patients with Chronic Granulomatous Disease.

### Clinical protocol

**Fig 1:**
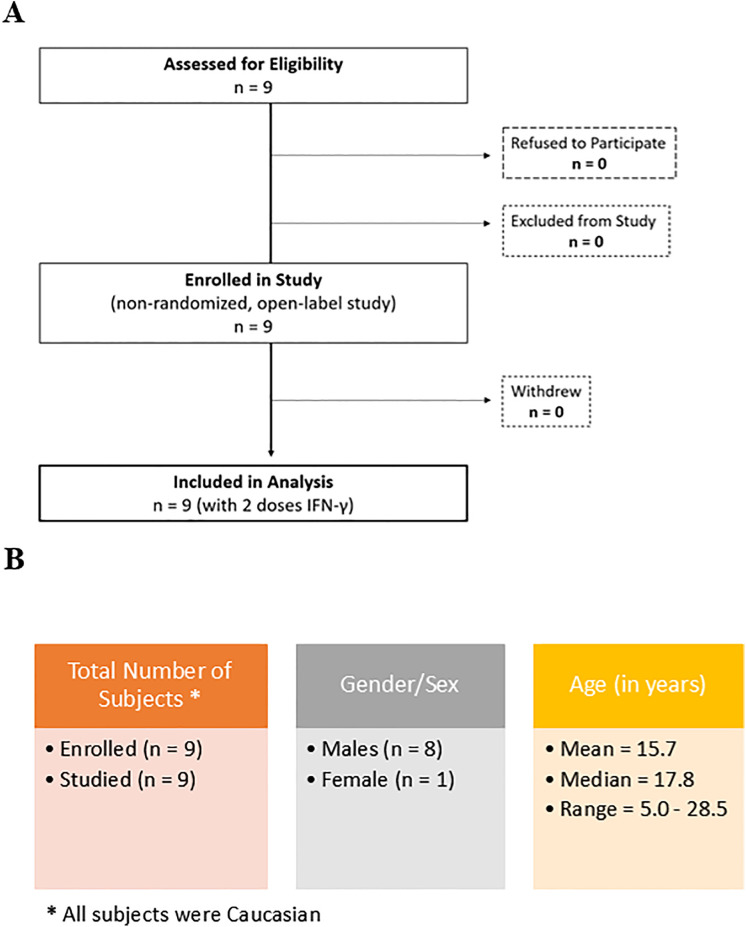
panel A. Flow diagram showing recruitment, enrollment, and analysis in the clinical trial. Panel B. Demographics for the patients enrolled in the study.

Nine patients with CGD, 8 male and 1 female, were eligible and enrolled on the study (Fig 1A), after confirming normal blood counts and a negative pregnancy test in the female of childbearing age. Demographic, biochemical/molecular, and genetic information about the patients is included in Fig 1 B and Table 1. At the time of the study, seven patients were on regular triple prophylaxis including Actimmune^®^ (Interferon-gamma 1-b), Bactrim, and Itraconazole. Two (CGD 07 and 09) who had refused chronic administration of Actimmune^®^ were taking the prophylactic anti-bacterial and anti-fungal medications noted above. For patients on regular IFN-γ administration, the drug was stopped for a week to allow a drug-washout period. Prophylactic antibiotics and antifungal medications at standard doses were given daily to all nine patients during the protocol. All subject patients gave a blood sample before the 1st dose of IFN-γ (*off IFN-γ*). IFN-γ administration was initiated at the dose of 50 µg/m^2^ given subcutaneously on Monday, Wednesday, and Friday in the evening before bedtime. Then, 10–12 hours after the 1st dose, and the 4th dose of IFN-γ additional blood samples were obtained. The study extended from March 1, 2018, until August 31, 2020, although the first patient was not enrolled until after July 6, 2018.

**Table 1:** Demographic information, molecular and genetic defects of CGD patients.

*Patient*	*Age (yrs)*	*Gender*	*Western Blot Analysis*	*Gene/Chromosome*	Mutation		
					*DNA Change*	*Protein Change*	*Variant*
CGD 01	28.5	Male	gp91^Phox^, p22^Phox^ not detected	*CYBB*/ X	hemizygous c.1A > G *	p.Met1?	Start Loss Mutation
CGD 02	18.6	Male	gp91^Phox^, p22^Phox^ not detected	*CYBB*/ X	hemizygous c.1461 + 1G > T	N/A	Splice Site Mutation
CGD 03	17.9	Male	gp91^Phox^, p22^Phox^ not detected	*CYBB*/ X	hemizygous c.804 + 1G > T	N/A	Splice Site Mutation
CGD 04	12	Male	gp91^Phox^, p22^Phox^ not detected	*CYBB*/ X	hemizygous c.1682_1712del31	p.Val561AspfsX5	Nonsense Mutation
CGD 05	5.3	Male	gp91^Phox^, p22^Phox^ not detected	*CYBB*/ X *IFNGR1*/ 6	hemizygous c.1253C > A heterozygous c.566C > A	p.Ser418Tyr p.Thr189Lys	Missense Mutation
CGD 06	23.2	Male	gp91^Phox^, p22^Phox^ not detected	*CYBA*/ 16 *CYBA*/ 16 *CYBB*/ X	heterozygous c.204-2A > T * heterozygous c.203 + 2T > C * hemizygous c.335C > A *	N/A N/A p.Ser112Tyr	Splice Acceptor Mutation Splice Donor Mutation Missense Mutation
CGD 07	17.8	Female	p47^Phox^ not detected	*NCF1*/ 7	homozygous c.75_76del	p.Tyr26Hisfs*26	Nonsense Mutation
CGD 08	5	Male	gp91^Phox^, p22^Phox^ not detected	*CYBB*/ X	hemizygous c.1314 + 2dupT	N/A	Splice Site Mutation
CGD 09	13.4	Male	gp91^Phox^ not detected	*CYBB*/ X	hemizygous c.456T > A	p.Tyr152X	Nonsense Mutation
	Mean Age = 15.7				* Derived from RNA sequence analysis		
	Median Age = 17.8						

Initial diagnostic evaluation of patients included NADPH oxidase activity by SOD inhibitable cytochrome c reduction; Western blot analysis for gp91^*phox*^, p22^*phox*^, p47^*phox*^, and p67^*phox*^; confirming X-linked carrier mothers with nitroblue tetrazolium (NBT) or dihydrorhodamine (DHR) tests; and mutational analysis by DNA sequencing (subjects 2–5 and 7–9) or derivation from RNAsequence (*RNAseq*) analysis (patients 1 and 6). Note that patient 06 has two *CYBA* mutations and one *CYBB* mutation. Of note was the fact that the mother of subject 06 had a normal DHR analysis with a single peak after her neutrophils were stimulated with PMA, suggesting a possible spontaneous mutation for the defect in *CYBB*.

No serious adverse events occurred during the study. No infections were noted in any of the patients while they were off IFN-γ. Non-serious adverse events of IFN- γ found in one patient after two doses of the drug included mild headache after administration which resolved rapidly with medication. Expected minor adverse events associated with phlebotomy were seen in three patients presenting as mild dizziness treated symptomatically with oral fluids. All patients who had previously been treated with IFN-γ returned to standard treatment with this drug at the end of the study.

### Experimental procedures

#### Cell isolation.

Neutrophils were isolated from heparinized peripheral blood by Dextran sedimentation, Ficoll-Hypaque density centrifugation, and hypotonic lysis of red blood cells [22,23]. Neutrophils were quantitated manually in a hemocytometer in the presence of 2% paraformaldehyde. Cells were 95–99% neutrophils and > 98% viable by trypan blue exclusion.

#### Cell function assays.

**Chemotaxis** was determined in response to Krebs Ringers Phosphate Dextrose (KRPD) buffer alone, N-formylmethionyl-leucyl-phenylalanine (fMLF, 0.1 µM), platelet activating factor (PAF, 0.4µM), and complement component 5 activated (C5a, 0.01 µM) with 1µM calcein-AM (Cat# C3100MP, Life Technologies, Carlsbad, CA) labeled neutrophils moving across fluorescence blocking PET membrane in Corning HTS FluoroBlok plates (Cat# 351162, Corning Life Sciences, Tewksbury, MA). Fluorescence was measured in a POLARstar Omega luminometer (BMG Labtech, Cary, NC) every 2 min at 37 °C for 1 hr, and the results presented as corrected fluorescence/min as previously described [24].

**Ingestion** was measured with Alexa488-*S. aureus* (Wood strain, 2 mg/ml) particles (Cat# S23371, Molecular Probes, Eugene, OR) opsonized in the presence of 20% pooled human serum for 30 min at 37°C with an assay adapted from a previously described protocol [25]. Neutrophils (10^6^) were incubated with opsonized bacteria (0.2 mg/ml) at 37°C. At Time 0 and every 5 min thereafter, 10^5^ cells were removed, placed at 4°C, washed twice with buffer, and resuspended in 0.1% trypan blue to eliminate attachment fluorescence [26]. Fluorescence of the resuspended pellet was documented in the POLARstar Omega luminometer (485/520 nm, Abs/Em) and presented over time up to 15 min.

**Bactericidal activity** was determined against *Staphylococcus aureus (S. aureus)* (Cat# 25923, American Type Culture Collection [ATCC], Manassas, VA) with 1:1 ratio of neutrophils to bacteria in 10% normal human serum by standard colony count technique [22–24]. The results were represented as % viable bacteria at various times over the incubation period.

**Formation of neutrophil extracellular traps (NETs)** was measured in neutrophils in the presence of either PMA or the *S. aureus* [27,28]. Briefly, 0.5x10^5^ PMN were incubated in a 96 well plates in the presence of either media alone (RPMI plus 0.025% Human Serum Albumin and 10mM HEPES, pH7.4) or KRPD buffer and 40nM PMA, for 3.5 hours at 37°C in the presence of SYTOX Green (2.5µM, Cat# S7020, Molecular Probes, Eugene, OR), a nucleic acid- binding stain unable to penetrate competent membranes. NETs formation was determined from the extracellular fluorescence measurement from wells in presence and absence of SYTOX Green and PMA after the incubation. Results are presented as the ratio of stimulated to non-stimulated fluorescence. Alternatively, *S. aureus* was expanded for 3.5 hours until log phase, pelleted and quantified by spectrometry. Bacteria were then rotated for 90 minutes, at 37°C, together with neutrophils (10^6^) at a ratio of 10:1 in the presence of 10% human serum. Aliquots containing 0.5 x 10^5^ PMN were placed into a 96-well plate and fluorescence measured in the presence and absence of 2.5µM SYTOX Green. NETs formation was quantified in presence and absence of S. *aureus* and presented as the ratio of stimulated to non-stimulated fluorescence.

#### Biochemical assays.

**Superoxide anion** was measured in intact cells as superoxide dismutase (SOD)-inhibitable cytochrome c reduction in response to fMLF (1 µM) and PMA (200 ng/ml) as previously described [22–24].

**Expression of CD11b** was determined after incubation of neutrophils (5 x 10^5^) with buffer, PMA (200 ng/mL), or fMLF (1µM) for 5 minutes at 37°C. Samples were quenched with KRPD buffer on ice and centrifuged at 300g for 10 minutes at 4°C. Pellets were resuspended with 20 mL phycoerythrin-labeled mouse anti-human CD11b or isotype control–labeled mouse anti-human IgG2a (Cat# 347557, Cat# 349053, respectively, BD Biosciences, Milpitas, CA) in 80 mL KRPD and incubated on ice in the dark for 30 minutes. After another wash and centrifugation, cells were fixed with 2% paraformaldehyde. Cell surface CD11b was detected with direct immunofluorescence using a Yeti 5-laser Analyzer (Propel Labs, Fort Collins, CO), with 10 000 events counted, and expressed as fluorescence, as described [22–24]. Analysis of the neutrophil population determined that 94% to 98% of cells were positive for CD11b.

For **F-actin content**, neutrophils, as above, were stimulated with buffer, PMA, or fMLF in Hanks’/HEPES buffer (pH 7.15) with 0.05% human serum albumin (HSA) for 10 minutes at 37°C. Cells were permeabilized and fixed with 0.01% lysophosphatidylcholine/3.7% formaldehyde for 5 minutes at 37°C and labeled with 0.165 mM NBD (N-(7- nitrobenz-2-oxa-1,3-diazol-4-yl)phallacidin, NBD-phallacidin, Cat# N354, Molecular Probes, Eugene, OR) in HSA for 10 minutes at 37°C with shaking. After centrifugation at 400g for 6 minutes at 4°C, pellets were resuspended in ice-cold HSA. Fluorescence was measured by flow cytometry as above and presented as fluorescence [22–24].

**MHC Class II antigen expression** on the neutrophil surface including HLA-DR, CD274, and CD40) was assayed by flow cytometry. Briefly, neutrophils (10^6^ cells) were resuspended with Zombie yellow (Cat. #423103, Biolegend, San Diego, CA) and compensation beads (Cat# 01-2222-41, Life Technologies, Eugene, OR) in FACS buffer (PBS, pH 7.4, 0.5% BSA, and 2 mM EDTA); washed once; resuspended in FACS buffer with PE anti-human HLA-DR (Cat# 307606), APC anti-human CD274 (Cat# 329708), Brilliant violet 421 anti-CD40 (Cat# 334332), or FITC anti-CD45 (Cat# 304006, all Biolegend, San Diego, CA); and incubated for 30 min at 4°. Cells were fixed with 4% paraformaldehyde in PBS buffer washed again and resuspended in FACS buffer. After two additional washing steps, the cells were resuspended and read in a flow cytometer as above counting 10,000 events per marker. The neutrophil population was selected with side scatter and CD45 marking and analysis via flow cytometry using a Yeti 5-laser Analyzer. The live selected cells were analyzed for HLA-DR, CD40, and CD274 expression. A small low side scatter population has been noted as contamination. To reduce the influence of this population on the change in expression in the neutrophils, a tight live neutrophil gate was applied to each sample prior to the traditional light scatter gate. Back gating from the low side scatter brightly stained events to the scatter plot showed that the population was most likely contaminating monocytes or B lymphocyte cells that were not fully excluded by the selection process. The remaining contamination was between 2–5% for each sample after analysis. The first 3 patient sample sets collected were only stained for each individual marker (HLA-DR, CD40, CD274) plus a fixable live/dead exclusion dye (Zombie Yellow). Subsequent sample sets were stained with the live/dead exclusion dye, CD45, and the 3 markers of interest. The change in method did not affect the changes in expression between the off IFN-γ and after 4th dose samples. As all the neutrophils have a baseline level of HLA-DR, CD40, and CD274, Median Fluorescent Intensity (MFI) best represents the changes in the data between the two conditions.

#### Cell lysates.

At sampling time points, cell lysates were produced from neutrophil preparations after centrifugation and resuspension at 10^8^ cells/mL in lysis buffer containing a protease inhibitor cocktail (Cat# 05892970001, Roche, Mannheim, Germany) and frozen at −70°C [22–24].

**Nitric oxide (NO) levels** in neutrophil lysates were inferred using a colorimetric assay to measure the combined nitrate and nitrate levels (Cat# STA-802, Cell Biolabs, San Diego, CA) [5]. Protein concentrations in the cell lysates were measured using the Pierce BCA Protein assay kit (Cat# 23225, ThermoFisher Scientific, Waltham, MA) and nitrite plus nitrate levels were normalized to these values.

**Western blot analysis** of lysates (50 µg protein) was completed with resolution of proteins on 10% or 15% SDS-PAGE and blotting onto nitrocellulose membranes as is described in detail [5,7,8,22–24,29,30]. Detection of proteins on membranes was performed with exposure to primary antibodies as follows, for FCGR1A (Cat# TA506343, Origene, Rockville, MD), FCGR1B (Cat# PA526205, Invitrogen, Waltham, MA), GCH1 (Cat# MA527276, Life Technologies, Carlsbad, CA), gp91^*phox*^ (Cat# SC130543, Santa Cruz Biotechnology, Santa Cruz, CA), MD2/LY96 (Cat# MA515766, Invitrogen, Waltham, MA), p47^*phox*^ (Cat# ab181090, Abcam, Cambridge, UK), and GAPDH (Cat# TA802519, Origene, Rockville, MD). Secondary antibodies included Goat anti-Mouse IgG Horse Radish Peroxidase (Cat# 10004302, Cayman Chemical, Ann Arbor, MI), and Goat Anti-Rabbit IgG H&L Horse Radish Peroxidase (Cat# ab6721, Abcam, Cambridge, UK) and Donkey anti-Goat IgG H&L Horse Radish Peroxidase (Cat# ab6885, Abcam, Cambridge, UK). Immune complexes were then detected with markers from Amersham (Cat# GERPN800E, Cytivia GE Healthcare, Marlborough, MA) an enhanced chemiluminescence system (Cat# 34580, Life Technologies, Carlsbad, CA). Chemiluminescence was analyzed using the G: BOX Chemi XL1.4 Fluorescent & Chemiluminescent Imaging System and CAM-GX-CHEMI-XL1.4 Camera Lens Assembly (Syngene, Cat# 05-GBOX-CHEMI-XL1.4 (G:BOX Chemi XL1.4)/ Cat# 05-CAM-GX-CHEMI-XL1.4, Frederick, Maryland, USA) and associated software (Genesys software version 1.8.2.0, Syngene, Frederick, Maryland, USA). Quantitation of proteins detected was done with densitometry using ImageJ software (http://imagej.nih.gov/ij/). Western blots contained samples from an individual patient’s lysate samples off IFN-γ and after the 4th dose of IFN-γ. Quantitation of the densitometry measurements for specific proteins was completed by calculating the ratio of the specific time after administration by the result from off IFN-γ. This represented the fold change and results were summarized as mean ± SEM for the number of separate measurements completed.

#### Gene expression studies: next generation RNA sequencing (*RNA-Seq*).

RNA from neutrophils was isolated by standard technique [5] and used to prepare libraries using the Universal Plus mRNA-seq kit from Tecan Genomics, Redwood City, CA. The mRNA template libraries were sequenced as paired 150 bp reads on the Illumina NovaSeq 6000 platform at the University of Colorado’s Genomics and Sequencing Core. Derived sequences were analyzed with gSNAP, Cufflinks and R for sequence alignment and determination of differential gene expression [31,32]. Reads were mapped to the human genome (GRCh38 by gSNAP), expression derived by Cufflinks, and differential expression analyzed with a paired t-test in R [33]. Differentially expressed genes were significant at p < 0.05. The raw gene expression data from this study have been deposited into Array Express database at EMBL-EBI (https://www.ebi.ac.uk/fg/annotare/login/) under accession number E-MTAB-13674.

### Statistical methods

As limited data existed at the initiation of our current study for *in vivo* neutrophil gene expression, function, and biochemical data from CGD patients that were measured off and after administration of IFN-γ, we used our previous *in vivo* adult study [5] that determined gene expression, NO generation, and protein levels to power this study. The difference in these parameters for subjects in that study was at least 2.5-fold of the common standard deviation. With 9 subjects serving as their own control, the current study would provide 95% power to detect a difference of 1.3 common standard deviations between off and post-IFN-γ treatment with a two-sided paired t-test and a significance level of 0.05. We recognized that individual samples are more variable, yet using the same subject as his own control reduces variability, and we believed that 9 subjects for the current studies would provide interpretable results.

Study outcomes included: chemotaxis with buffer, fMLF, PAF, and C5a; ingestion of fluorescently labeled *S aureus*; bactericidal activity against *S. aureus*; PMA and fMLF stimulated O_2_^-^ production, quantitation of various proteins by Western blot; CD11b surface expression and F-actin assembly after stimulation with buffer, PMA, and fMLF; and surface expression of MHC class II proteins, HLA-DR, CD274, and CD40. Also measured were nitrate plus nitrite (NO production) in cell lysates, NETs formation, and gene expression off IFN-γ and after administration of the cytokine. Differences in levels of each outcome after administration of the cytokine were compared to results for off IFN-γ for each subject using paired t-tests as noted above.

Repeated measurements analysis of variance (RM ANOVA) was used to evaluate whether gene expression significantly changed off IFN-γ compared to after administration. As each subject’s off IFN-γ baseline was inherently different, the expression level at this condition was subtracted from the post-administration condition to determine their differences and this difference from baseline was then used for RM ANOVA as described above in RNA techniques. The significance for each gene was required to be at a level of p < 1.76e-6 (i.e., p < 0.05 with strict Bonferroni correction for 28463 genes). A 1.25-fold cutoff in gene expression was used as a practical change for purposes of this analysis. For genes significant at the 1.25-fold cutoff, a heatmap was generated showing the average change in expression across all subjects for each sampling.

Microsoft Excel for Microsoft 365 (64-bit) with “Analysis ToolPak” software was used for all analysis except gene expression. The overall significance level was set at 0.05. Except for the previously mentioned Bonferroni correction for the gene expression analysis, adjustments for multiple comparisons were not performed, as this was a pilot study of exploring IFN-γ in human subjects. The emphasis of the results is on the effect sizes.

## Results

### Patients enrolled

Eight males and one female with CGD but no recent history of infections were enrolled in the study. A clinical protocol summary and patient characteristics are included in Fig 1 and Table 1.

### Neutrophil function assays

**Fig 2:**
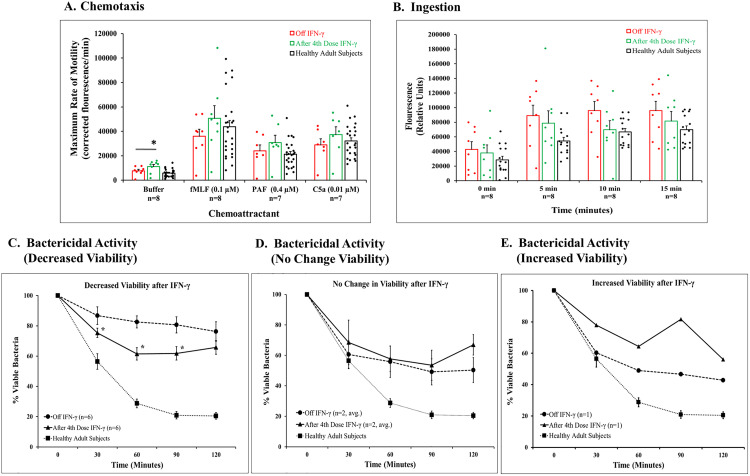
Neutrophil function assays completed on CGD patients off and after the 4^th^ dose of IFN-γ. **A. Chemotaxis** was measured using transmembrane migration technique in response to buffer, fMLF, PAF, and C5a with concentrations noted. Fluorescence -labeled patient cells were detected as they crossed fluorescence-blocking PET membrane into lower wells of the system. Fluorescence was measured as described in Methods and expressed as maximum rate of fluorescence/min. Results are presented as a bar graph with dot plot overlay of patient data, red for patients off IFN-γ; green for patients after 4th dose; and black for healthy adult subjects (n = 25) for comparison. Bars and brackets represent mean ± SEM with numbers of patients for each stimulus studied noted below. *Denotes difference between patients off IFN-γ and after 4th dose p = 0.037, paired, 2-tailed t test. **B. Ingestion** was determined with serum opsonized Alexa488 *S.aureus* particles and fluorescence of washed PMNs measured at various times noted below and results presented as a bar graph with dot plot overlay of patient data. Bar and patient designation are as stated in **A.** Bars and brackets represent mean ± SEM with the number of subjects studied below. Results for healthy adult subjects (black, n = 15) are included for comparison. **C, D, and E. Bactericidal Activity** was determined against *S. aureus* with 1:1 ratio of neutrophils to bacteria in 10% normal human serum by standard colony count technique, and results were represented as % viable bacteria at various times over the 2-hour incubation period. **2C** shows results for six patients demonstrating decreased viability after administration of IFN-γ. Symbols and bars represent mean ± SEM for six patients (circles, off IFN-γ; triangles, after 4th dose). Results for healthy adult subjects (n = 11, squares) are included for comparison. *Indicates significance between off IFN-γ and after 4th dose at each time point, p = 0.045 or less by paired, 2-tailed t test. **2D** illustrates average of results for two patients off IFN-γ and after the 4th dose showing no change in viability, and **2E** presents results for a single patient with increased viability after administration of IFN-γ with the same symbols as shown for **D.**

**Fig 2A** summarizes results for chemotaxis of fluorescently labeled neutrophils. Random migration with buffer alone and directed migration to fMLF, PAF, and C5a were increased after 4th dose of IFN-γ compared with off IFN-γ; but only results for buffer were significant. Results for 25 healthy adults are shown for comparison.

Ingestion of Alexa 488-labeled *S. aureus* by patients’ neutrophils was robust in the absence of IFN-γ and reached a maximum by 5 min. No further changes were seen at 10 and 15 mins (Fig 2B). Results after the 4th dose of IFN-γ were lower at various times. Studies for neutrophils from healthy adult subjects are included for comparison. Increased phagocytosis by CGD neutrophils has been shown previously [34,35].

Killing of serum-opsonized *S. aureus* by IFN-γ-exposed neutrophils from 6 patients (Fig 2C) was significantly increased at 30, 60, and 90 minutes compared to killing with their cells off IFN-γ. Results for healthy adult subjects are also shown. Three patients (CGD 01, 05, and 08) exhibited no improvement in bacterial killing after IFN-γ treatment (Figs 2D & 2E). Patient CGD 05 had a concurrent mutation in *IFNGR1* (discussed below).

**Fig 3:**
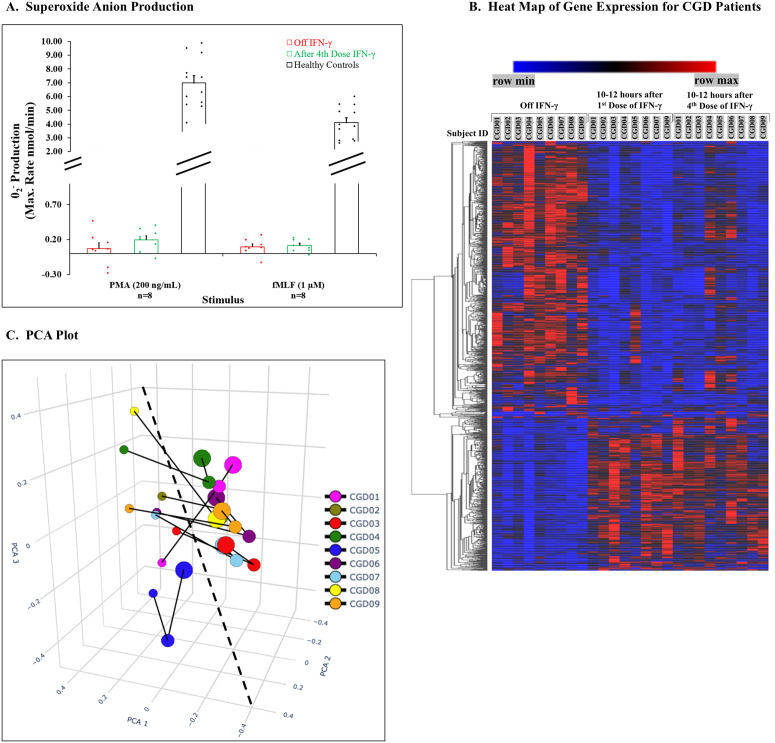
Neutrophil Superoxide anion production, Heat map for gene expression from neutrophils of CGD patients, and PCA plot for genome-wide gene expression. **3A. Superoxide anion production** was measured as SOD inhibitable cytochrome c reduction in response to PMA and fMLF as noted. Results are presented as bar graphs with dot plot overlays for patient data expressed as nmol/min. Bars and brackets represent mean ± SEM with the number of patients studied below. Results off IFN-γ are red, and after the 4th dose, green. Results for healthy adult subjects (black, n = 12) are included for comparison. **3B. Heat map** showing changes for all significant genes between off IFN-γ and 10-12 hours after the 1st and 4th dose (50 µg/m^2^) of the cytokine given on a routine schedule noted in Methods. Gene expression for each gene is provided for each patient according to treatment noted at the top of the graph. The cells are colored according to expression level relative to row (i.e., relative within gene). Genes that responded to IFN-γ with increased expression are at the bottom and those with decreased expression are at the top. **3C. PCA plot** of global gene expression in three dimensions for neutrophils from CGD patients showing off IFN-γ and after 1st and 4th doses. Colored circles define an individual subject’s results with the smallest circle representing global gene expression off IFN-γ; the middle size representing gene expression after the 1st dose of the cytokine; and lastly, the largest representing expression after the 4th dose. The solid lines link results for specific patient’s results. The dashed line provides a visual aid to separate off IFN-γ and after administration results. Note that subject 05 values are clustered closer together suggesting a smaller, but non-negative effect of the drug. In contrast, results for subjects 08 and 09 demonstrate greater effects than the remainder of the patients in the cohort.

Despite improved bactericidal activity in six patients, neutrophils from only three patients produced even minimal amounts of O_2_^-^detected by cytochrome c reduction after the fourth dose of IFN-γ (Fig 3A). There was no relationship between O_2_^-^ release and bactericidal activity.

### Gene expression studies

We evaluated neutrophil gene-expression in CGD patients off IFN-γ and 10–12 hours after administration of the 1st and 4th doses in a standard clinical-prophylactic regimen. Results for gene expression are presented in Fig 3B as a heatmap showing changes for genes with a > 1.25-fold increase or decrease in expression off IFN-γ compared to 10–12 hours after the 1st dose (1162 genes changed, 771 decreased and 391 increased) and 10–12 hours after the 4th dose (869 genes changed, 483 decreased and 386 increased).

Functionally relevant neutrophil genes that were altered by IFN-γ administration for the nine patients are shown in Table 2. Two sets of columns represent comparison of gene expression off IFN- γ compared to 10–12 hours after the 1st and 4th IFN-γ doses and include the fold change and p-value. Genes directly related to neutrophil function and number, including NADPH oxidase components (*CYBB*, *NCF1*), *FCGR1A*, *FCGR1B*, and innate immune receptors (*Ly96*, *NOD1*, and c-type lectins, *CLEC5A*, *6A*, and *9A*) were increased significantly after both doses of IFN-γ. *CLEC4A* was increased only after the 4th dose of IFN-γ. Exceptions to this included *TLR5* and *TLR8*, which did not increase. Similar changes were demonstrated for genes not generally expressed by neutrophils, including those for MHC Class II and MHC Class I proteins. Gene expression for guanylate binding proteins (GBPs) was also induced by IFN-γ and showed significant results for *GBP1–7*. Proteins for these genes provide diverse immune supportive functions [5]. In addition, a novel protein, apolipoprotein L3, which was recently shown to have antimicrobial activity in non-immune barrier cells [4], also demonstrated increased expression in CGD neutrophils after administration of IFN-γ. A modest but significant increase in the expression for the C-X-C Motif Chemokine Receptor 4 was seen after the 4th dose of IFN-γ.

**Table 2:** Relevant neutrophil genes altered by IFN-γ administration to CGD patients (n = 9).

	Off IFN-γ Vs. After 1st Dose				Off IFN-γ Vs. After 4th Dose			
Gene	Control Mean	Treatment Mean	Fold Change	p-value*	Control Mean	Treatment Mean	Fold Change	p-value *
**MHC (HLA) Class II**								
*CD274*	72.52	145.76	2.01	0.00926	66.37	128.94	1.94	0.00931
*CD40*	0.82	2.81	3.43	0.00258	0.84	5.17	6.15	0.00386
*CD74*	937.14	2249.39	2.4	0.00011	916.03	2637.93	2.88	1.9E-05
*CIITA*	8.77	19.43	2.22	0.0128	8.42	20.91	2.48	0.00167
*HLA-DMA*	16.24	56.97	3.51	0.00039	17.56	82.84	4.72	3.7E-05
*HLA-DMB*	13.73	58.27	4.24	0.00126	14.58	79.74	5.47	7.9E-05
*HLA-DOB*	4.53	5.55	1.23	0.59177	4.53	4.42	−1.02	0.08117
*HLA-DPA1*	37.95	54.55	1.44	0.08936	40.35	100.29	2.49	0.00587
*HLA-DPB1*	35.36	42.86	1.21	0.51023	38.97	90.26	2.32	0.01675
*HLA-DQA2*	0.43	0.72	1.67	0.11489	0.4	1.39	3.47	0.09005
*HLA-DQB1*	23.34	78.39	3.36	0.00046	27.44	121.34	4.42	0.0002
*HLA-DQB2*	1.02	2.42	2.37	4.1E-05	1.21	5.19	4.29	0.00508
*HLA-DRA*	119.92	287.24	2.4	0.00472	126.25	446.8	3.54	0.00088
*PDCD1LG2*	7.24	21.19	2.93	0.00085	6.5	23.97	3.69	0.00072
*RFX5*	10.07	26.89	2.67	0.00019	9.95	29.15	2.93	2E-06
**MHC (HLA) Class I**								
*PSMB8*	364.44	475.83	1.31	0.00973	354.23	469.3	1.32	0.00245
*PSMB9*	721.21	1057.29	1.47	0.00305	692.71	996.86	1.44	0.0019
*TAP1*	665.1	1083.33	1.63	0.00269	646.76	1016.7	1.57	0.00078
*TAP2*	232.7	529.8	2.28	0.00126	232.7	481.67	2.07	0.07613
**Guanylate Binding Proteins**								
*GBP1*	491.98	1146.36	2.33	0.00361	465.88	1026.84	2.2	0.00127
*GBP1P1*	6.66	45.52	6.83	0.0003	6.33	50.67	8	6.2E-05
GBP2	830.26	1163.06	1.4	0.01622	789.43	1077.31	1.36	0.00696
*GBP3*	35.56	97.14	2.73	0.00098	36.15	90.22	2.5	0.00053
*GBP4*	90.9	359.09	3.95	0.00056	89.43	297.58	3.33	4.7E-05
*GBP5*	1066.71	2743.46	2.57	0.00465	991.64	2754.55	2.78	0.00143
*GBP6*	6.72	20.53	3.06	0.00051	6.2	23.6	3.81	0.00013
*GBP7*	0.22	1.16	5.27	0.02849	0.3	1.23	4.1	0.0121
**Innate Immune Receptors**								
*CLEC4D*	47.74	64.97	1.36	0.27616	46.87	67.58	1.44	0.02332
*CLEC5A*	3.73	5.89	1.58	0.00213	4.08	7.62	1.87	0.00737
*CLEC6A*	0.25	0.77	3.08	0.00027	0.28	0.98	3.5	0.00079
*CLEC9A*	2.44	6.17	2.53	0.00011	3.13	5.38	1.72	0.01094
*LY96*	305.74	396.1	1.3	0.00635	288.84	339.73	1.18	0.00574
*NOD1*	8.64	17.7	2.05	0.00149	8.28	16.58	2	0.00057
*TLR5*	47.58	28.89	−1.65	0.0049	44.51	24.29	−1.83	0.02653
*TLR8*	156.79	153.64	−1.02	0.77992	154.73	150.13	−1.03	0.7162
**Fcγ Receptors**								
*FCGR1A*	298.56	604.84	2.03	0.02166	271.8	542.03	1.99	0.00943
*FCGR1B*	134.06	209.42	1.56	0.05263	122.5	182.62	1.49	0.03577
**NADPH Oxidase Proteins**								
*CYBB*	86.85	211.75	2.44	0.00059	79.28	200.3	2.53	1.3E-05
*NCF1*	1085.19	1999.71	1.84	0.00085	1103.95	1920.73	1.74	0.00026
**GTP Cyclohydrolase 1**								
*GCH1*	21	88.16	4.2	0.0002	20.44	89.63	4.39	4.4E-05
**C-X-C Motif Chemokine Receptor 4**								
*CXCR4*	576.41	720.09	1.25	0.10682	581.58	742.95	1.28	0.01059
**Apolipoprotein**								
*APOL3*	10.01	58.17	5.81	3.27E-05	9.86	43.77	4.44	0.00018378

*GCH1* was significantly upregulated over four-fold (Table 2) after both treatments with IFN-γ; and because it is the first and rate-limiting enzyme in the biopterin pathway providing tetrahydrobiopterin, a cofactor for nitric oxide synthetase (NOS) activity, it has the potential to contribute to increased NO generation. It is noteworthy that gene expression for NOS 1,2, and 3 was not changed from baseline levels after administration of IFN-γ (see data in Array Express database, Methods). This was also true for adult subjects treated with IFN-γ [5].

Blood samples from the CGD patients were obtained 10–12 hours after a dose of IFN-γ. In healthy adults, the peak of gene expression occurred between eight and 12 hours after IFN-γ administration [5]. Thus, it is possible that other genes reaching the criteria for increase were underreported because of sample timing. Detailed kinetic studies could not be practically obtained in our patients. Regardless, gene expression for the CGD patients that we studied was very similar to that induced by IFN-γ in healthy adults [5].

Analysis of gene expression presented a few issues about the patient cohort. First, *RNA-seq* analysis demonstrated a mutation in CGD 05 affecting 50% of the reads aligned to interferon-gamma receptor 1 (*IFNGR1*), which would result in a change in the protein sequence p. Thr189Lys. Sequencing genomic DNA from the patient’s cells confirmed a heterozygous missense mutation in *IFNGR1* (See Table 1), exon5, c.566C > A. Although this mutation has been listed in association with susceptibility to mycobacterial disease due to *IFNGR1* deficiency (MedGen UID 863300), this variant has not been reported in individuals affected by *IFNGR1*-related conditions. Furthermore, advanced modeling and biophysical properties of the missense variant did not lead us to believe that the variant could disrupt the resultant *IFNGR1* protein; and CGD 05 did not have clinical complications that might be expected with *IFNGR1* deficiency. This suggested that the variant was of uncertain clinical significance. No other patient in this cohort demonstrated mutations in *IFNGR1*, and no mutations were noted in *JAK1* or *2*, *STAT1*, or *IFNGR2* in any subject in the cohort, including CGD 05.

The global gene-expression patterns off IFN-γ compared with those after administration of the cytokine varied among the nine patients (Fig 3C and S1 Fig). PCA plots for global gene expression for specific patients showed clustering of results around sampling time-points for off IFN-γ compared with those after the 1st and 4th dose. Results for CGD 05 were clustered more closely together as well as more closely to the off-IFN-γ sample, demonstrating a smaller response to IFN-γ; patients CGD 08 and CGD 09 were more widely separated with larger responses between off IFN-γ and IFN-γ administrations. While suggesting a heterogeneity not related to the molecular type of CGD, these results could not provide a rationale for further segregating the cohort into different groups.

### Biochemical studies

Neutrophil chemotactic response is a complex process that requires expression of cell-surface adhesion molecules such as CD11b/CD18 and dynamic assembly of the actin cytoskeleton [13–15]. We examined cell-surface levels of CD11b as well as the extent of filamentous actin content in patient neutrophils before and after IFN-γ by flow cytometry.

**Fig 4:**
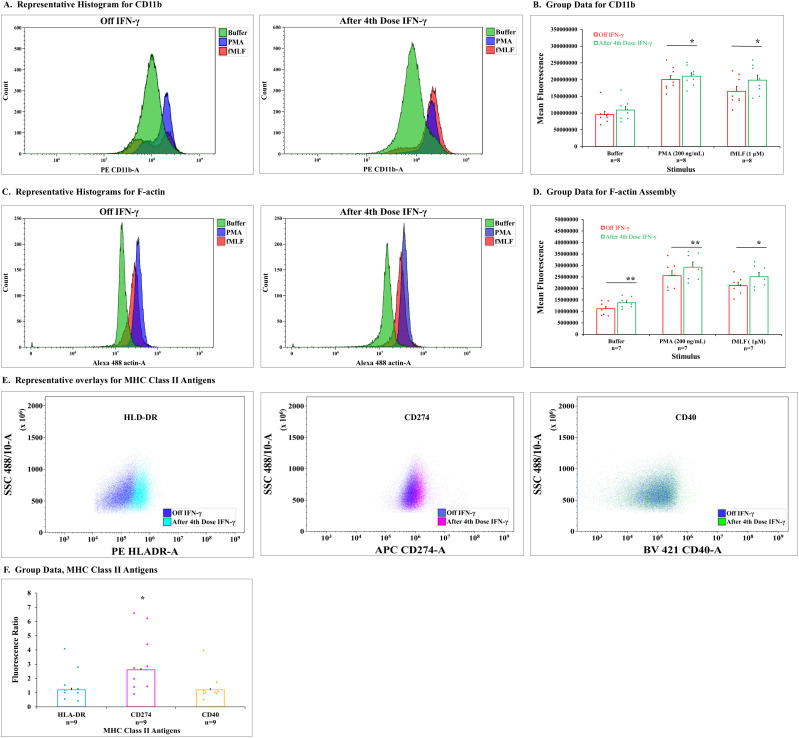
Flow cytometry measurement of CD11b, F-actin expression and content, and MHC Class II antigen expression of neutrophils from patients with CGD. **A. Flow cytometry histograms showing representative examples of CD11b** expression for patient neutrophils off IFN-γ (left panel) and after the 4th dose (right panel) after incubation with buffer (green), PMA (200 ng/ml, blue), and fMLF (1µM, red). Plots are cell counts vs fluorescence. **B. Group results for CD11b expression.** Results are presented as bar graphs with dot plot overlays of patient data. Mean fluorescence is plotted by stimulus with bars and brackets representing mean ± SEM and number of patients studied noted below. Red bars and symbols represent off IFN-γ results and green bars and symbols represent after 4th dose with * denoting significant difference, p ≤ 0.031, paired, 2-tailed t test. **C. Flow cytometry histograms showing representative examples of F-actin content and assembly** for patient neutrophils off IFN-γ (left panel) and after the 4th dose (right panel) after incubation with buffer (green), PMA (200ng/ml, blue), and fMLF (1µM, red). Plots are cell counts vs fluorescence. **D. Group results for F-actin content and assembly.** Results are presented as bar graphs with dot plot overlays of patient data. Mean fluorescence is plotted by stimulus with bars and brackets representing mean ± SEM and number of patients studied noted below. Red bars and symbols represent off IFN-γ and green bars and symbols represent after 4th dose with * denoting significant difference, p = 0.017 and ** difference, p ≤ 0.0013, paired, 2-tailed t test. **E. Representative results for MCH Class II expression.** Flow cytometry histogram overlays showing representative examples for expression of HLA-DR, CD274, and CD40 off IFN-γ (blue) and after 4th dose of IFN-γ on left, middle, and right, teal, pink, and green respectively. **F. Group results for MHC Class II expression**. Ratio of fluorescence expression after 4th dose to off IFN-γ are presented for HLA-DR (teal), CD274 (pink), and CD40 (orange) as bar graphs with dot plot overlays of patient data. Bars and brackets represent mean ± SEM of ratios with number of patients studied below. *Denotes a significant difference p = 0.0034, paired, 2-tailed t test.

Fig 4A shows histograms of CD11b expression from a representative patient off IFN-γ (left panel) and after the 4th dose (right panel) as stacked plots of number of cells vs. log fluorescence, and Fig 4B summarizes results for the group. PMA and fMLF increase the expression of CD11b regardless of the IFN-γ status. For patients as a group, CD11b expression after stimulation with PMA and fMLF was slightly but significantly increased after the 4th dose of IFN-γ (Fig 4B).

Similar histograms for F-actin content in a representative patient off IFN-γ and after the 4th dose of IFN-γ (left and right panels, respectively) are shown in Fig 4C. Both PMA and fMLF increased the F-actin assembly off and after IFN-γ administration. Summary results for the patient group (Fig 4D) showed F-actin content was significantly increased in resting cells and with either stimulus after the 4th dose of IFN-γ.

To further confirm *RNA-seq* studies, we evaluated expression of MHC Class II antigens, HLA-DR, CD274, and CD40 by flow cytometry. Composite flow scans for HLA-DR, CD274 and CD40 off IFN-γ and after the 4th dose from samples of a representative patient are shown in Fig 4E (left, center, and right, respectively). Although a shift with increased antigen-expression is seen after administration of IFN-γ for all antigens, the most dramatic change occurred with CD274. Results for the entire group of patients expressed as the ratio of Mean Fluorescent Activity (MFI) off IFN-γ to after the 4th dose of the cytokine are shown in Fig 4F. Ratios were increased for all antigens, but only CD274 was significant.

**Fig 5:**
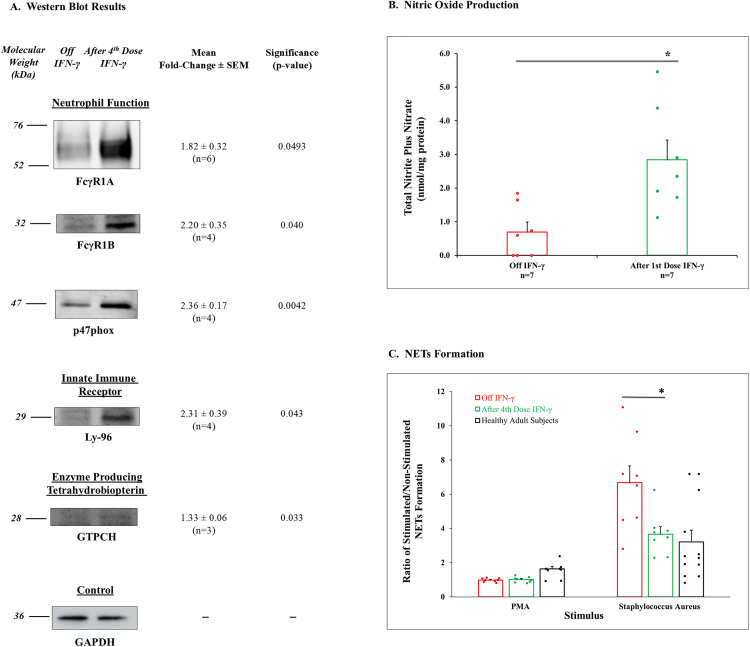
Representative Western blots for proteins from specific gene groups, nitric oxide production, and NETs formation. **A. Representative Western blots and quantitation for the proteins** as fold-change of scanned results after administration of IFN-γ compared to off the cytokine are presented with mean ± SEM, number of patient lysates evaluated, and significance. **N.**B. Since all patients except for CGD07 had *CYBB* and/or *CYBA* mutations, gp91^*phox*^ and p22^*phox*^ were not detected on Western blot analysis, and results are not shown. CGD 07 had a normal appearance of gp91^*phox*^ and p22^*phox*^ on Western blot. The housekeeping enzyme glyceraldehyde-3-phosphate dehydrogenase (GAPDH) was included as a loading control. **B. NO generation** presented as Total Nitrate plus Nitrite in PMN lysates (nmol/mg protein). Results are presented as bar graphs with dot plot overlays of patient data. Assays were completed off IFN-γ (red bars and symbols) and after 1st dose of the cytokine (green bars and symbols). *Denotes difference between off IFN-γ and after 1st dose, p = 0.002 by paired, 2-tailed t test. **C. Neutrophil Extracellular Traps (NETs) formation** was measured by the fluorescence of extracellular Sytox Green as described in Methods and is represented as the ratio of stimulated to non-stimulated NETs formation after incubation of CGD PMNs with PMA (n = 8) or incubation with *S. aureus* (n = 8) vs. buffer control. Results are presented as bar graphs with dot plot overlays of patient data. Assays were completed off IFN-γ (red bars and symbols) and after 1st or 4th dose of the cytokine (green bars and symbols). Healthy adult subject results are shown with black bars and symbols bars when appropriate for comparison. Bars and brackets represent mean ± SEM with the number of samples studied below. *Indicates a difference between off IFN-γ and after the 4th dose, p = 0.033 by paired, 2-tailed t test. Results for PMNs from healthy adult subjects (n = 10 exposed to PMA and n = 12 exposed to *S. aureus*) are included for comparison.

For additional confirmation of gene expression changes, we performed Western blot analysis on neutrophil lysates from patients for representative proteins for specific gene groups. Fig 5A shows representative blots and quantitation of proteins as fold-change for the available lysates studied. Content of proteins for *FCGR1A*, *FCGR1B*, and *NCF1* were significantly increased after the 4th dose of IFN-γ. Since all but one patient had mutations affecting *CYBB* and/or *CYBA*, and protein levels for gp91^*phox*^ could not be detected, blots are not shown for that protein. *LY96* and *GCH1* were also significantly increased after the 4th dose.

Increased expression of *GCH1* and NO production was found in neutrophils from healthy adult subjects treated with IFN-γ [5]. Since *GCH1* gene expression and protein (GTP cyclohydrolase 1, GTPCH) content were increased in CGD neutrophils after administration of IFN-γ (Table 2, Fig 5A), we measured the presence of nitrite/nitrate as a proxy for the unstable NO molecule in cell lysates from CGD patients. NO in cell lysates was increased after the 1st dose (Fig 5B) compared to off IFN-γ. Generation of NO by IFN-γ could provide an alternative mechanism to support bactericidal activity in patients with CGD.

#### Neutrophil extracellular traps (NETs).

Neutrophils control microbial pathogens by an additional process called NETosis using neutrophil extracellular traps or NETs [36]. Composed of processed chromatin associated with selected granular and cytosolic proteins and peptides, NETs are effective in killing extracellular organisms; but they are a double-edged sword because they can promote inflammation and autoimmunity [37]. Results after induction with PMA, the commonly used NETs stimulus, off IFN-γ and after the 4th dose of the cytokine, are presented in Fig 5C, represented as ratio of stimulated/unstimulated cells. CGD patient values were below the range for normal subjects (1.5–3.0) and were unchanged off IFN-γ and after the 4th dose, perhaps representing another abnormality in microbicidal activity. NETs formation associated with ingestion of *S. aureus* compared to its absence was also evaluated (Fig 5C). The ratio was increased in CGD patients off the cytokine; and after the 4th dose, the ratio decreased, moving significantly toward normal levels exhibited by healthy subjects. This may balance the deficient contribution of oxidant-induced NETosis by CGD neutrophils. Reduction of ingestion-induced NETs by IFN-γ may serve to reduce inflammatory and autoimmune complications of CGD.

## Discussion

Administration of IFN-γ reduces the risk of bacterial infection in patients with CGD [17] and can prolong life in some patients in association with restoration of modest activity of the phagocytosis-associated respiratory burst [12]. We sought to determine whether IFN-γ administration might also promote non-oxidative mechanisms that could contribute to improved protection against infection, and by using CGD as an experiment of nature, to gain greater understanding of the biologic effects of IFN-γ in the immune response to infection. As background for evaluation of CGD patients, we studied first the effect of IFN-γ administration on neutrophils from normal, healthy adults [5]. Detailed measurement of *in vivo* genome-wide changes in mRNA before and after treatment with IFN-γ provided clues for how this cytokine might influence neutrophil activity in CGD patients. The gene list from the current patient study identified relationships to known neutrophil functions organized (Table 2) into protein families or groups of genes associated with classical as well as less-well-appreciated aspects of neutrophil function, indicating that IFN-γ induces extensive changes in the neutrophil phenotype.

We found that non-directed migration was increased after administration of IFN-γ in most of our patients. Directed movement in response to chemotactic stimuli was increased for the group, but this did not reach significance. Ingestion of fluorescently labeled *S. aureus* by neutrophils from the patients was robust, as previously shown [34,35], but this activity was not significantly different after administration of IFN-γ. Two thirds of the patients exhibited a significant increase in phagocytic killing of *S. aureus* after the 4th dose of IFN-γ; no increase was shown in two patients. For one patient, bacterial viability increased after IFN-γ. He was found to have a coexisting mutation in IFNGR1, but it is not clear that this mutation affected neutrophil function.

Gene expression for two high-affinity IgG receptors, (*FCGR1A* and *FCGR1B*) was upregulated by IFN-γ in this study, an effect that has clear implications for enhanced phagocytic clearance. However, in our assay system IFN-γ administration did not further increase the robust baseline level of phagocytosis. Increased transcription of several innate immune receptors was induced by IFN-γ treatment. These molecules, including *LY96* which forms the LPS receptor, *NOD1*, and *CLEC4D-9A*, trigger changes involved in pathogen clearance. Finally, IFN-γ caused transient increases in the expression of *CYBB* and *NCF1*, which encode specific components of the respiratory burst enzyme. IFN-γ could induce transcription of mutated genes but fail to induce production of a normal protein, resulting in an incomplete oxidase complex and classic absence or blunting of respiratory burst activity of CGD. This possibility was supported by Western blot analysis (Fig 5A) and blunted oxidase activity (Fig 3A).

Our data demonstrate that IFN-γ induces expression of numerous genes not generally recognized to be regularly expressed by neutrophils. Many genes involved in the MHCII system are upregulated after IFN-γ administration in CGD. Other studies support the concept that neutrophils can acquire antigen-presenting capacity like that characteristic of adaptive immunity [38–41]. Several components of the MHC Class I system were upregulated in our study; and as suggested by other studies [42–46], acquisition of antigen processing by the MHC Class I system might be another host-defense benefit induced in neutrophils by IFN-γ and an expression of collaboration between the innate and adaptive immune systems.

Upregulation of gene expression for *GBPs* and *APOL3* were also induced by IFN-γ in CGD patients. Antimicrobial roles for many of these classes of molecules have been described [4,47,48]. Increased delivery of microbicidal peptides into phagolysosomes might partially compensate for compromised oxidant-mediated killing. Finally, *GCH1* was also upregulated following IFN-γ administration. This protein is the first and rate-limiting enzyme in the biopterin pathway, generating an essential cofactor(s) for NOS enzymes, providing an alternative bactericidal pathway [49,50] that may be especially important in patients with CGD.

Two important issues are of note here. First, the timing of patient blood samples to 10–12 hours after the dose of IFN-γ could influence gene expression and other results presented in this study. In adults, detailed kinetic analysis found peak responses at 8–12 hours after administration of IFN-γ [5]. Obtaining multiple, timed samples was not possible in our study. Our results for CGD patients at a single time could possibly have underestimated gene expression.

Second, one of the patients enrolled (CGD 05) was found in the *RNA-seq* analysis to have a mutation in the *IFNGR1* gene, and DNA analysis confirmed the mutation (Table 1). Biochemical modeling and biophysical analysis suggested that this mutation should not have had a damaging effect on receptor function. No other mutations in signaling pathways for IFN-γ were discovered in this or any other patient in the cohort. Global gene-expression analysis demonstrated a smaller, but not a negative response to IFN-γ by this patient’s cells (Fig 3C, and S1 Fig), indicating that this was not a null mutation. Results from two other patients (CGD 08 and CGD 09), in contrast, exhibited larger responses to IFN-γ-induced gene expression (Fig 3C, S1 Fig) without an apparent genetic or biochemical basis. These results indicate a heterogeneity of response to IFN-γ in patients with a primary genetic syndrome that could affect individual clinical responses.

Biochemical processes that link gene expression and the functional activities of neutrophils were affected by IFN-γ administration. Neutrophil chemotaxis is a complex process that requires expression of cell-surface adhesion molecules, including CD11b/CD18, and dynamic assembly of the actin cytoskeleton [13–15]. Actin assembly but not CD11b expression was increased by IFN-γ in CGD cells that were not further stimulated. Stimulation by either PMA or fMLF enhanced both CD11b expression and F-actin assembly in CGD neutrophils.

The change in bactericidal activity noted in two thirds of the patients studied was not associated with improvement in phagocyte oxidase activity and production of O_2_^-^ (Fig 3A) in response to administration of IFN-γ. Two issues are of note. First, our assay for O_2_^-^ production was not the sensitive measurement described by Kuhns et al [12], and we might not have been able to detect the same levels of residual NADPH oxidase activity that were described in that report. However, most of our patients had gp91^phox^ deficiency with splice site, nonsense, and start-site mutations and would be in the lowest quartile of residual oxidant production in that study. Of the two autosomal patients, one was a male with homozygous p22^phox^ deficiency and a coexistent missense mutation of gp91^phox^and not expected to have significant residual O_2_^-^ production. Only one other had p47^phox^deficiency.

However, an alternative process could support bactericidal function. *GCH1* expression and protein (GTP cyclohydrolase, GTPCH) content (Table 2 and Fig 5A) were increased after administration of IFN-γ implicating increased production of NOS cofactor and resultant activity. Enhanced generation of NO was demonstrated after IFN-γ in PMN lysates from CGD patients (Fig 5B). Because NO, and reactive nitrogen intermediates (RNI) produced from it, are broad-spectrum microbicidal agents and have been shown to be an alternative to oxidant mediated killing in neutrophils [50], we suggest that IFN-γ mediated increases in RNI might provide CGD neutrophils with at least a partial replacement for the loss of reactive oxidant generation.

Three representative MHC Class II antigens demonstrated increased expression in CGD neutrophil lysates after administration of the IFN-γ; and, in one, CD274, the differences were significant. These findings correlated with gene expression results and had implications for both innate and adaptive immune systems. Presentation of antigens by MHCII occurs on antigen-presenting cells (APCs), and neutrophils have not been thought to play a role in this process. However, studies have shown that neutrophils can express MHCII molecules that are functional and induce T-cell activation when treated with pro-inflammatory stimuli including IFN-γ [40–45]. Neutrophil expression of MHCII and acquisition of dendritic cell-like properties under culture conditions and at inflammatory lesions also challenge the concept that neutrophils can never express MHCII components or act as APCs [41]. Our findings indicate that CGD neutrophils could have APC activity and support the possibility that antigen presentation is an IFN-γ-induced contribution to its infective-protective effect when given to CGD patients. We propose that IFN-γ might induce operational MCHI and MCHII antigens in CGD neutrophils that could present microbial peptides to and could activate CD8-positive memory T-cells [43], an underappreciated function of the innate immune system and an expression of collaboration between innate and adaptive immune systems.

Neutrophils fight pathogens by an additional process, generation of NETs [36]. Neutrophil NETs production when stimulated by PMA requires cell release of reactive oxidants; and, therefore, PMA has no effect on NETs formation in CGD cells. However, production of NETs was induced by ingestion of *S. aureus* which does not require oxidant generation. NETs formation was increased in CGD neutrophils off IFN-γ but returned toward normal levels after the 4th dose of the cytokine, balancing the deficient oxidant-induced pathway for NETosis. Excessive NETs formation has been linked to inflammatory, autoimmune, and other diseases [37,51] and may at least partially explain why CGD patients are predisposed to these complications. IFN-γ administration may thus reduce the risk of these disorders in treated CGD patients.

Administration of IFN-γ induces dramatic changes in circulating neutrophils from patients with CGD. The alterations in function, gene expression, and biochemical assays, demonstrate that the effects of this cytokine on neutrophils include enhancements in classically recognized neutrophil activity (i.e., random cell migration and motility, pathogen recognition by innate immune receptors, bactericidal activity, CD11b expression, and F-actin assembly) as well as largely unrecognized functional changes (i.e., interaction with the adaptive immune system via MHCI and MHCII, expression of microbicidal proteins including guanylate-binding proteins and apolipoprotein L3, and upregulation of NO production).

Our findings suggest that there may not be a single mechanism by which IFN-γ enhances the neutrophil host defense capacity, but rather several that could account for the clinical benefit of this cytokine. Although defective function of neutrophils, monocytes, and macrophages is central to the pathology of CGD, it is possible that IFN-γ also improved host protection in other components of the immune system beyond those we studied here. Nevertheless, we believe that our findings are important for understanding how the expanded use of this cytokine might support individuals with other disorders of compromised immune function.

## Supporting information

S1 Fig PCA plot of global gene expression for CGD patients in a HTML file. The organization and representation of the data for the figure is identical to Figure 3C, except for the absence of the dashed line.
(HTML)

S1 Data Western blot raw images for Figure 5. (PDF)

S2 Data List of Abbreviations in the Manuscript Text. (DOCX)

S3 Data Excel file of Raw Data for Figures 2–5. (XLSX)
