# Supplementary material for: Interferon-gamma 1b-induced gene expression alters neutrophil function in patients with chronic granulomatous disease
Source: PLoS One. 2025 Sep 8;20(9):e0331657. doi: 10.1371/journal.pone.0331657 (PMC12416707; doi:10.1371/journal.pone.0331657)
Supplement: S1 Data — (PDF) [file pone.0331657.s002.pdf]

Source Data:
Full Length Western Blots
shown in Figure 5A

FcγR1A

MW = Molecular Weight Markers
V2 = Off IFN-γ
V4 = 12 hours after 4th Dose of IFN-γ

MW V2 V4 MW

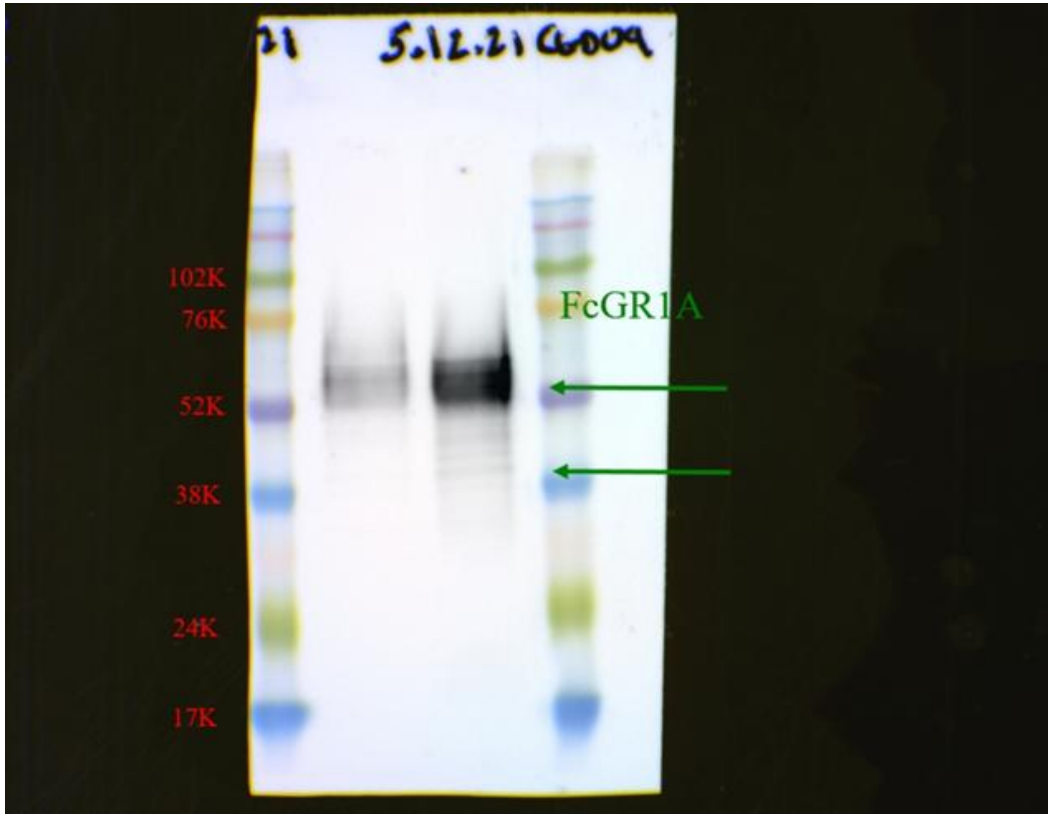

102 kDa
76 kDa
52 kDa
38 kDa
24 kDa
17 kDa

MW V2 V4 MW

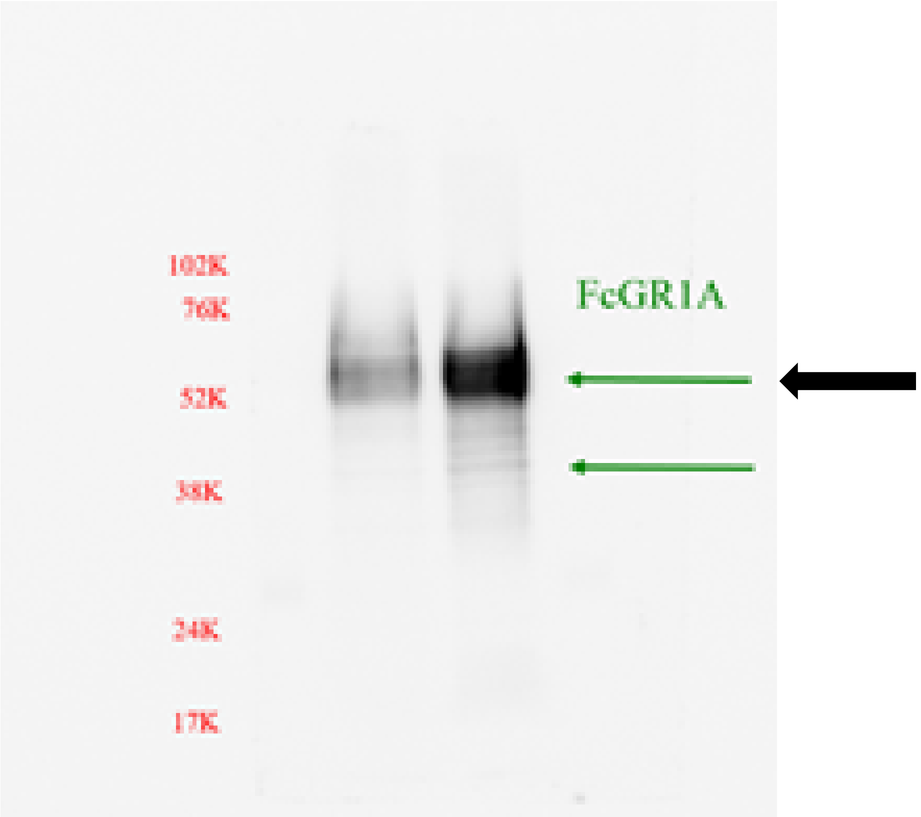

FcγR1A

Syngene Camera System/Genesys Software *(see methods)* rendering of original full blot provided to better show Molecular Markers in color.

Original chemiluminescence *(see methods)* full blot provided (used in Figure 5A).

Source Data:
Full Length Western Blots
shown in Figure 5A

FcγR1B

MW = **Molecular Weight Markers**
V2 = **Off IFN-γ**
V4 = **12 hours after 4th Dose of IFN-γ**

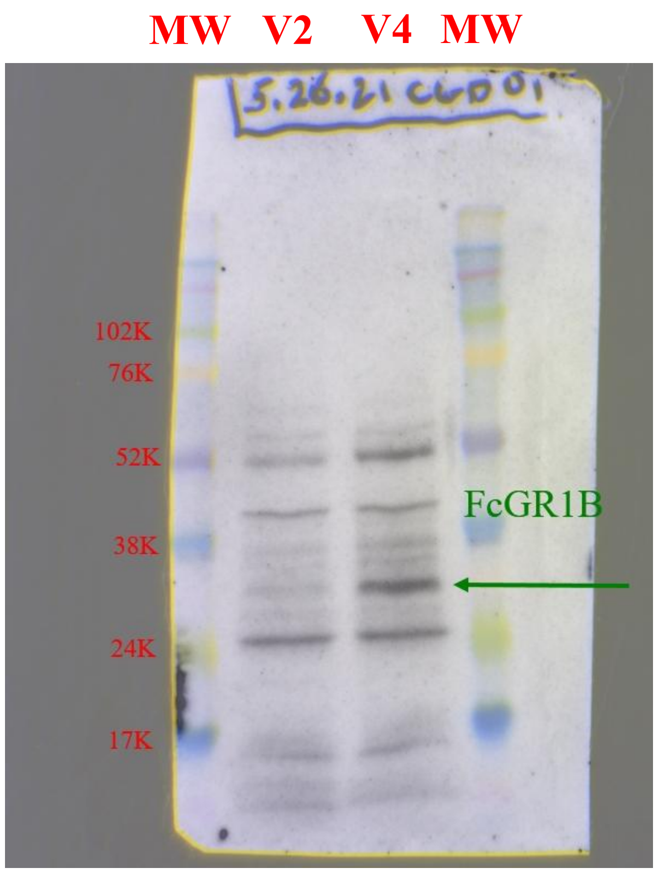

102 kDa
76 kDa
52 kDa
38 kDa
24 kDa
17 kDa

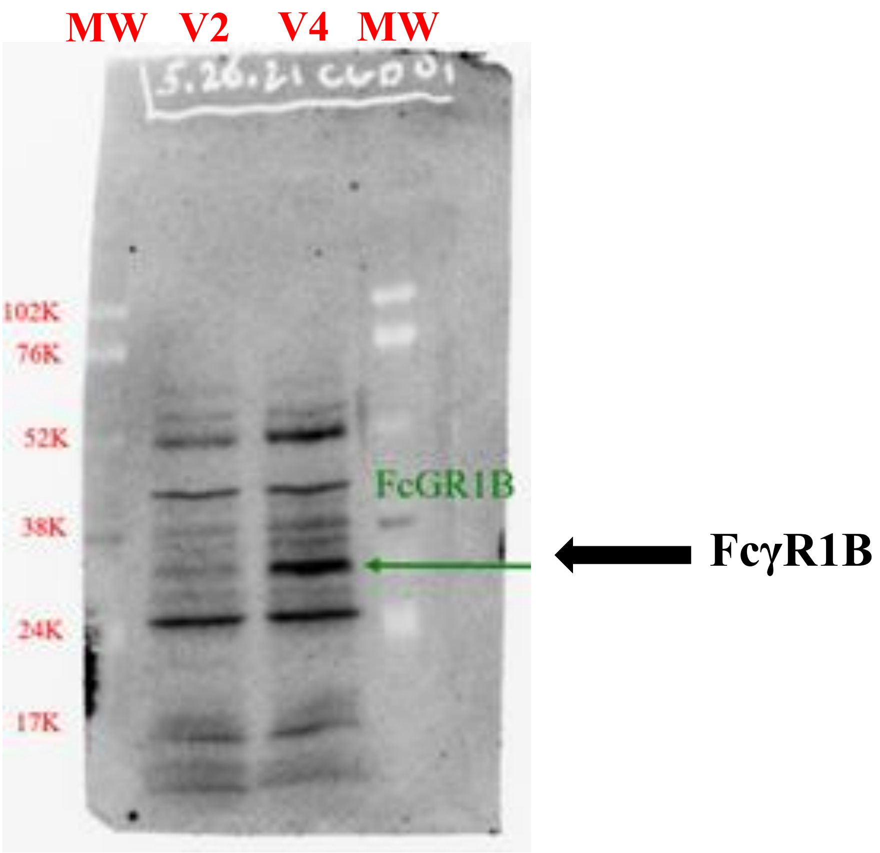

Syngene Camera System/Genesys Software *(see methods)* rendering of original full blot provided to better show Molecular Markers in color.

Original chemiluminescence *(see methods)* full blot provided (used in Figure 5A).

Source Data:
Full Length Western Blots
shown in Figure 5A

p47phox

MW = Molecular Weight Markers
V2 = Off IFN-γ
V4 = 12 hours after 4th Dose of IFN-γ

MW V2 V4

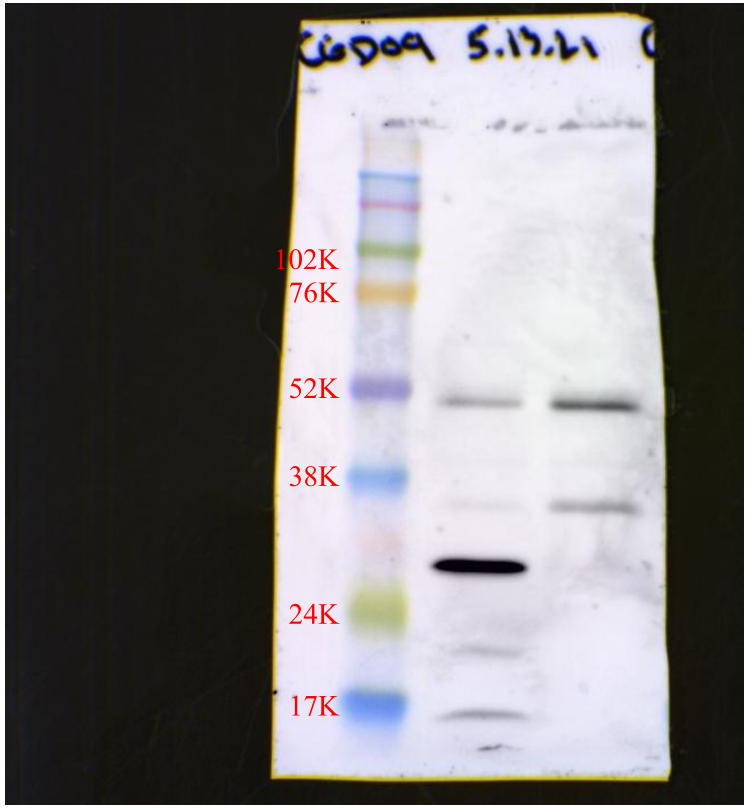

102 kDa
76 kDa
52 kDa
38 kDa
24 kDa
17 kDa

MW V2 V4

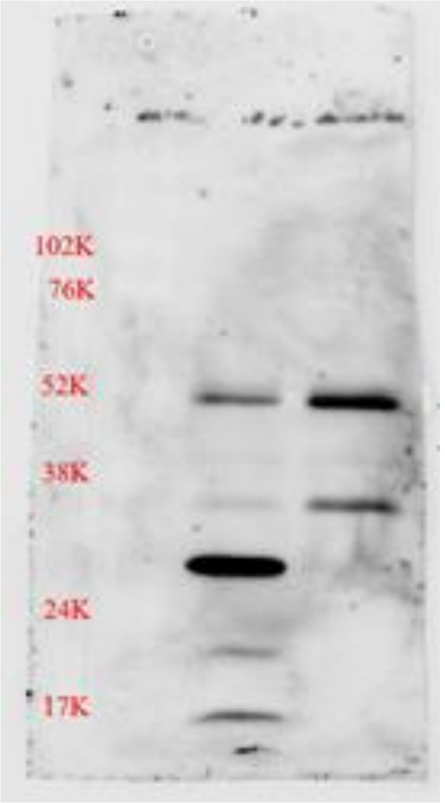

p47phox

Syngene Camera System/Genesys Software *(see methods)* rendering of original full blot provided to better show Molecular Markers in color.

Original chemiluminescence *(see methods)* full blot provided (used in Figure 5A).

Source Data:
Full Length Western Blots
shown in Figure 5A

Ly-96

MW = Molecular Weight Markers
V2 = Off IFN-γ
V4 = 12 hours after 4th Dose of IFN-γ

MW V2 V4

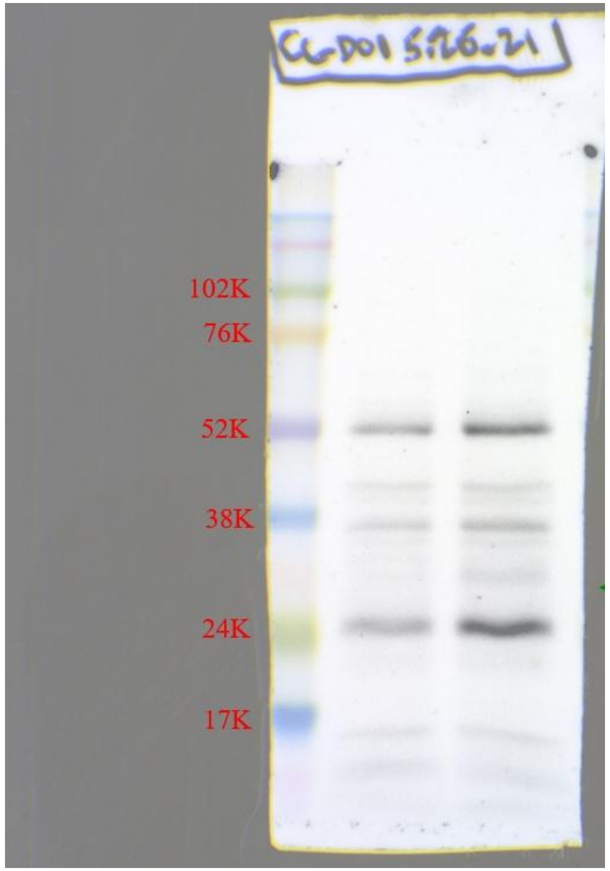

Syngene Camera System/Genesys Software *(see methods)* rendering of original full blot provided to better show Molecular Markers in color.

102 kDa
76 kDa
52 kDa
38 kDa
24 kDa
17 kDa

MW V2 V4

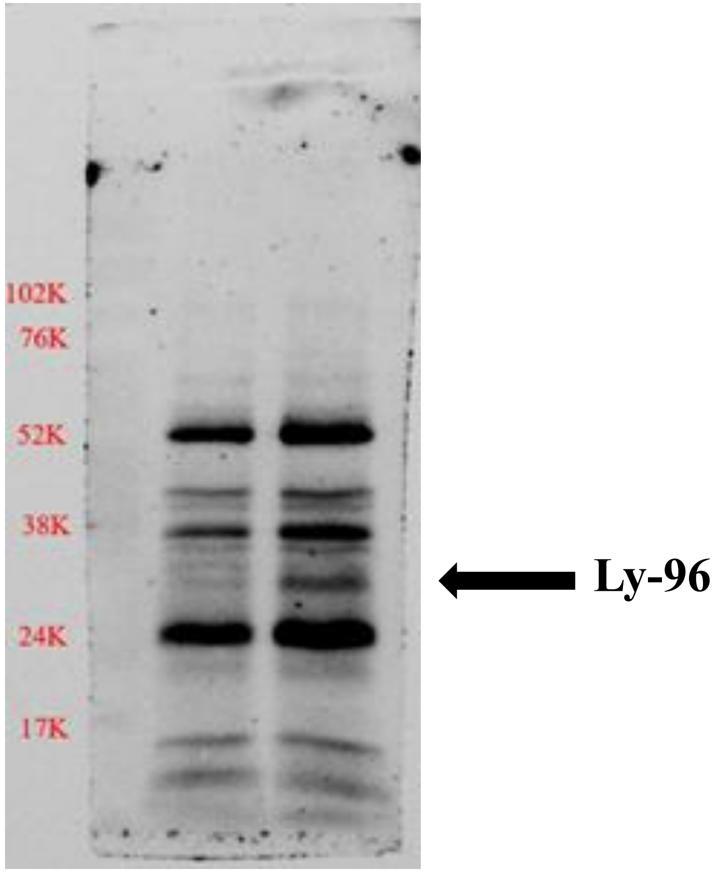

Original chemiluminescence *(see methods)* full blot provided (used in Figure 5A).

Source Data:
Full Length Western Blots
shown in Figure 5A

GTPCH

MW = Molecular Weight Markers
V2 = Off IFN-γ
V4 = 12 hours after 4th Dose of IFN-γ

MW V2 V4

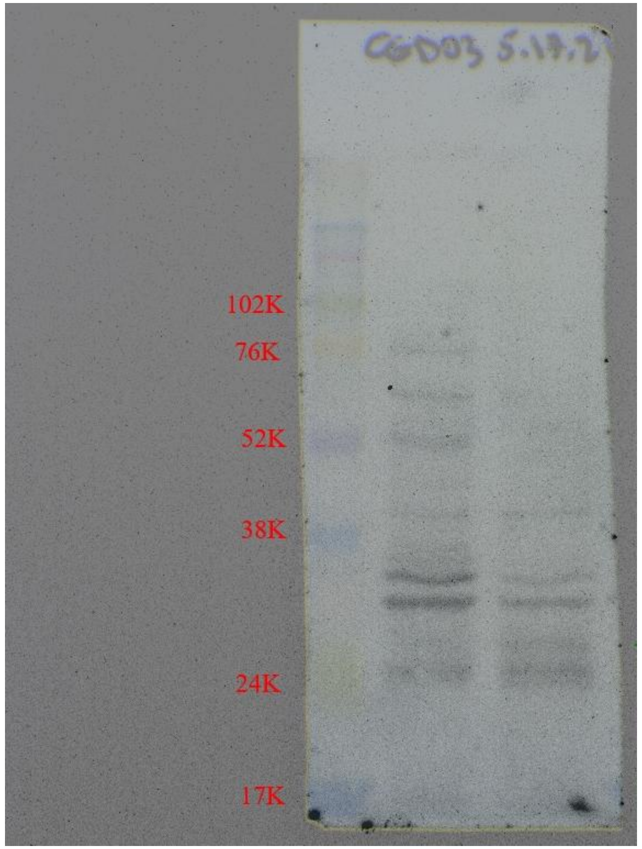

102 kDa
76 kDa
52 kDa
38 kDa
24 kDa
17 kDa

Syngene Camera System/Genesys Software *(see methods)* rendering of original full blot provided to better show Molecular Markers in color.

MW V2 V4

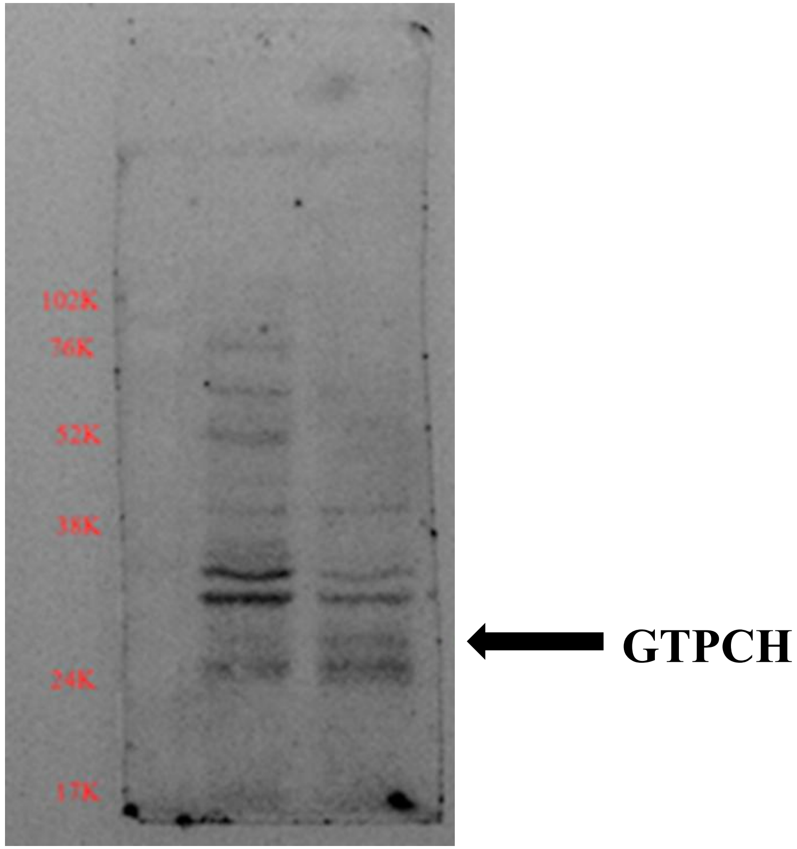

Original chemiluminescence *(see methods)* full blot provided (used in Figure 5A).

Source Data:
Full Length Western Blots
shown in Figure 5A

GAPDH

MW = Molecular Weight Markers
V2 = Off IFN-γ
V4 = 12 hours after 4th Dose of IFN-γ

MW V2 V4

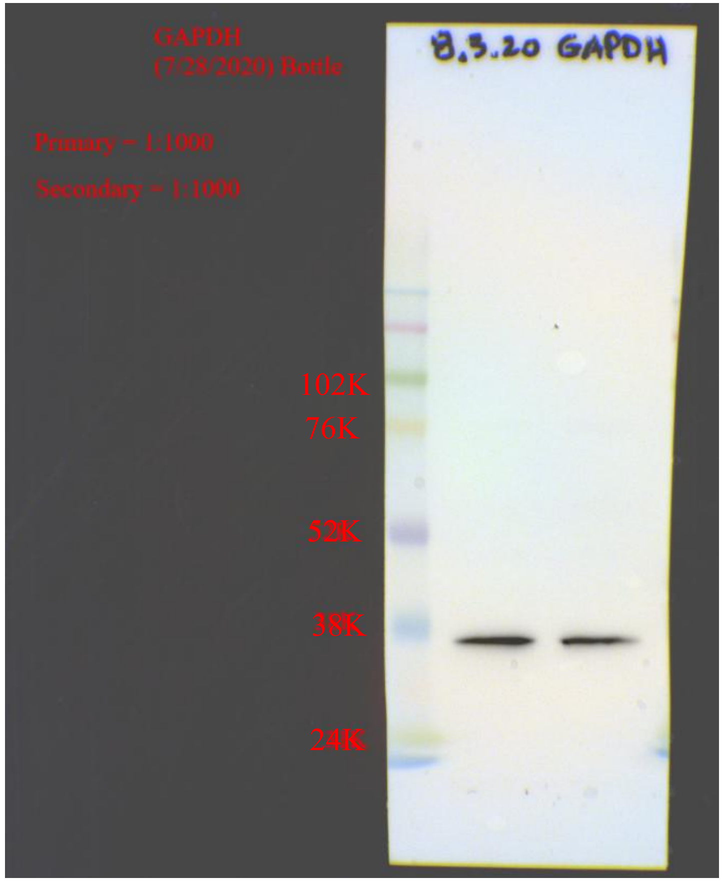

102 kDa
76 kDa
52 kDa
38 kDa
24 kDa

MW V2 V4

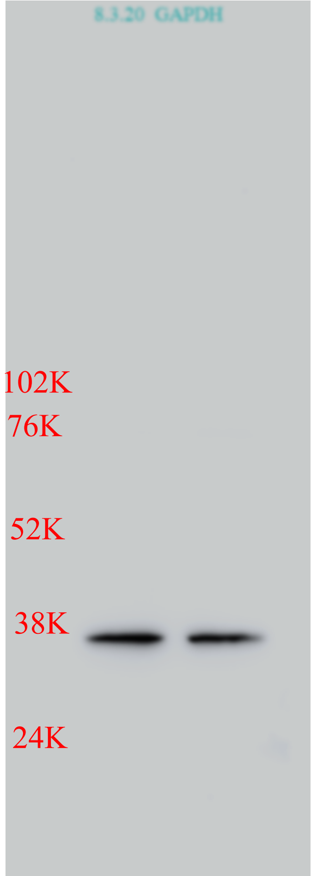

← GAPDH

Syngene Camera System/Genesys Software *(see methods)* rendering of original full blot provided to better show Molecular Markers in color.

Original chemiluminescence *(see methods)* full blot provided (used in Figure 5A).
