# Supplementary material for: Interferon-gamma 1b-induced gene expression alters neutrophil function in patients with chronic granulomatous disease
Source: PLoS One. 2025 Sep 8;20(9):e0331657. doi: 10.1371/journal.pone.0331657 (PMC12416707; doi:10.1371/journal.pone.0331657)
Supplement: S2 Data — (DOCX) [file pone.0331657.s003.docx]

**Supplementary Information (S3). List of Abbreviations in the Manuscript Text**

Full term Abbreviation

Antigen presenting cells (APCs)

Chronic granulomatous disease (CGD)

Complement component C5a (C5a)

Dihydrorhodamine (DHR)

Glyceraldehyde-3-phosphate dehydrogenase (GAPDH)

Interferons (IFNs)

Interferon-gamma or Interferon-gamma-1b (IFN-γ)

Interferon-gamma receptor (IFNGR)

Interferon-gamma receptor 1 or 2 (IFNGR1 or IFNGR2)

Human serum albumin (HSA)

Janus kinase 1 or 2 (JAK 1 or 2)

Krebs Ringers Phosphate with Dextrose (KRPD)

Neutrophil extracellular traps (NETs)

Nicotinamide adenine dinucleotide phosphate (NADPH)

N-Formyl-methionine-leucyl-phenylalanine (fMLF)

Nitric oxide (NO)

Nitric oxide synthase (NOS)

Nitroblue Tetrazolium (NBT)

Phorbol myristate acetate (PMA)

Phosphate buffered saline (PBS)

Platelet activating factor (PAF)

Randomized controlled trial (RCT)

Reactive nitrogen intermediate (RNI)

Signal transducer and activator of transcription 1 (STAT 1)

Staphylococcus aureus (S. aureus)

Superoxide anion (O_2_^-^)

Superoxide dismutase (SOD)
